# Positive interaction between melatonin and methyl jasmonate enhances *Fusarium* wilt resistance in *Citrullus lanatus*

**DOI:** 10.3389/fpls.2025.1508852

**Published:** 2025-05-01

**Authors:** Jingyi Yan, Tongshu Zhao, Yi Chen, Haiheng Liu, Chunhua Wei, Jianxiang Ma, Yong Zhang, Jianqiang Yang, Xian Zhang, Hao Li

**Affiliations:** ^1^ State Key Laboratory for Crop Stress Resistance and High-Efficiency Production, College of Horticulture, Northwest A&F University, Yangling, China; ^2^ Xi’an Modern Agricultural Science and Technology Exhibition Center, Xi’an Agricultural Technology Extension Center, Xi’an, China

**Keywords:** melatonin, methyl jasmonate, *Fusarium* wilt, FON2, watermelon

## Abstract

*Fusarium* wilt, caused by the soil-borne fungal pathogen *Fusarium oxysporum* (Fo), is widely recognized as one of the most devastating fungal diseases, inflicting significant damage on a wide range of agricultural and horticultural crops. Despite melatonin has recently emerged as a potential enhancer of plant resistance against Fo, the underlying mechanisms remain elusive. In this study, our results demonstrate that exogenous melatonin and MeJA enhance watermelon resistance against *Fusarium oxysporum* f. sp. *Niveum* race 2 (FON2) in a dose-dependent manner. The optimal concentration for melatonin and MeJA was determined to be 10 μM and 1 μM, respectively. Both melatonin and MeJA inhibited FON2 mycelial growth on PDA medium in a dose-dependent manner. Furthermore, exogenous melatonin significantly stimulated upregulation of MeJA synthesis genes and increased MeJA content upon FON2 infection. However, pretreatment with a MeJA synthesis inhibitor (DIECA) suppressed the induction of melatonin-induced resistance against FON2. Furthermore, MeJA also induced the upregulation of melatonin biosynthetic gene *caffeic acid O-methyltransferase 1* (*ClCOMT1*) and increased melatonin accumulation in response to FON2. Notably, the reduction in FON2 resistance caused by *ClCOMT1* deletion was completely restored through exogenous application of MeJA. These results suggest that melatonin facilitates MeJA accumulation, which provides feedback to promote melatonin accumulation, forming a reciprocal positive regulatory loop in response to FON2 infection. Additionally, polyphenol oxidase, phenylalanine ammonia lyase, and lignin are involved in the MeJA-induced resistance against FON2. The growing concern over minimizing pesticide usage and transitioning to sustainable and natural control strategies underscores the significant potential of such a mechanism in combating Fo.

## Introduction

Plants, as sessile organisms, constantly face a wide range of biotic and abiotic pressures throughout their lifespan. *Fusarium* wilt, caused by the soil-borne fungal pathogen *Fusarium oxysporum* (Fo), is a devastating disease with global distribution (Everts and Himmelstein, 2015). Fo is a facultative parasitic fungus capable of infecting plants and persisting in the soil. It ranks fifth among the top ten plant pathogenic fungi and exhibits wide host range infecting over 100 plant species (Dean et al., 2012). The primary mode of invasion for Fo is through apical intercellular spaces of root hairs or wounds, followed by colonization within vascular bundles where it obstructs them via secretion of cell wall lyases and mycotoxins resulting in browning of vascular tissues, leaf atrophy, gradual wilting, defoliation, and eventual death (Dean et al., 2012).

The immune system of plants has developed a sophisticated and efficient defense response network to combat infection by various pathogenic microorganisms. Within this network, jasmonic acid (JA) and its derivatives, such as methyl jasmonate (MeJA) and jasmonate-isoleucine complex (JA-Ile), play a pivotal regulatory role (Yang et al., 2019; Wang et al., 2021). In plants, the binding of JA to its receptor coronatine insensitive 1 (COI1) triggers the ubiquitination and degradation of jasmonate zinc-finger inflorescence meristem (JAZ) protein, thereby relieving the inhibition of MYC2 by JAZ and initiating the expression of disease-resistance related genes downstream of JA signaling pathway (Wang et al., 2021). In *Arabidopsis*, the mutation in JA-Ile synthesis gene *jasmonate resistant* (*jar*) *1–1* enhances plant susceptibility Fo infection (Trusov et al., 2009). Similarly, the sensitivity of tomato and *Medicago truncatula* to their respective Fo specialization types was increased by the mutations in JA synthesis genes *defenceless-1* (*def1*) and *lipoxygenases* (*lox*), respectively (Kavroulakis et al., 2007; Thatcher et al., 2016).

Melatonin (N-acetyl-5-methoxytryptamine) is a multifunctional bioactive molecule that is widely distributed in animals, plants, and microorganisms. Wei et al. (2018) discovered the first plant melatonin receptor CAND2/PMTR1 in *Arabidopsis* and elucidated its signaling mechanism for regulating stomatal movement, providing compelling evidence that plant melatonin functions as a novel plant hormone (Arnao and Hernandez-Ruiz, 2019). Numerous studies have demonstrated the pivotal role of melatonin not only in regulating plant growth, development, and abiotic stress responses (Arnao and Hernández-Ruiz, 2015; Onik et al., 2021), but also in conferring resistance to various fungal diseases including *Fusarium* wilt (Li et al., 2019a, b; Sun et al., 2019). For instance, exogenous application of melatonin has been shown to significantly induce resistance against *Fusarium* wilt in banana and cucumber (Wei et al., 2016; Ahammed et al., 2020). However, the signal transduction pathways and molecular mechanisms underlying melatonin-mediated regulation of plant *Fusarium* wilt resistance remain poorly understood. Melatonin interacts with MeJA to regulate plant defense responses against various biotic and abiotic stresses (Arnao and Hernández-Ruiz, 2018). However, the precise mechanisms underlying the crosstalk between melatonin and MeJA in regulating plant resistance to Fo remain elusive. Based on previous findings (Wei et al., 2016; Ahammed et al., 2020), we hypothesize that melatonin and MeJA synergistically regulate Fo resistance through reciprocal feedback loops.

The watermelon (*Citrullus lanatus*) holds significant global agricultural value, ranking among the top five fresh fruits with the highest consumption rates worldwide. Watermelon is a typical crop that exhibits intolerance to continuous cropping owing to its vulnerability to *Fusarium* wilt, caused by the specific infection of *Fusarium oxysporum* f. sp. *Niveum* (FON). With the emergence of mutated strains, the infectivity of FON varies from low to high. Currently, there are four internationally reported physiological races of FON, namely 0, 1, 2, and 3 (Zhou et al., 2010; Everts and Himmelstein, 2015). Among these races, FON1 and FON2 exhibit a global distribution (Hudson et al., 2021). FON1 is capable of infecting most watermelon varieties, while FON2 exhibits higher virulence and can infect nearly all watermelon varieties (Everts and Himmelstein, 2015). Additionally, FON produces thick-walled chlamydospores under natural conditions, displaying remarkable resistance to adverse environmental conditions and enabling prolonged survival in soil for over a decade (Zhang et al., 2015). Consequently, effective management of *Fusarium* wilt in watermelon poses an increasingly formidable challenge. In this study, we demonstrate a positive interaction between melatonin and MeJA in regulating FON2 resistance of watermelon.

## Materials and methods

### Plant materials and growth conditions

Watermelon [*Citrullus lanatus* (Thunb.) Matsum. & Nakai cv. Nongkeda No. 5] was utilized. The germinated seeds were sown in plastic pots containing a mixture of peat and vermiculite in a 1:5 (v:v) ratio. At the one-leaf stage, a group of 20 seedlings was transplanted into a container (40 × 25 × 15 cm) filled with Hoagland’s nutrient solution. The seedlings were grown in a greenhouse at Northwest A&F University with a temperature at 22-28/16-22°C (day/night), light intensity of 500 μmol m^−2^ s^−1^, and relative humidity of 65%–80%.

### Experimental design

In order to investigate the impact of exogenously applied melatonin and MeJA on watermelon’s resistance to FON2, different concentrations of melatonin and MeJA were added to the hydroponic nutrient solution for watermelon seedlings three days after transplantation. Melatonin and MeJA solutions were prepared as described previously (Li et al., 2021). The final concentrations of melatonin in the nutrient solution were set at 0, 1, 5, 10, or 50 μM. The final concentrations of MeJA in the nutrient solution were set at 0, 0.1, 1, or 10 μM. After a period of 24 h, a portion of the lateral roots were excised, and the remaining seedling roots were inoculated with FON2 spore suspension at a concentration of 1×10^6^ CFU/mL or distilled water (as control) for 15 minutes. Subsequently, the seedling roots were rinsed thrice with distilled water before being transplanted into hydroponic nutrient solution. To assess the involvement of MeJA in melatonin-induced FON2 resistance, diethyldithiocarbamic acid (DIECA, a JA biosynthesis inhibitor) were used to block MeJA biosynthesis (Li et al., 2021). The final concentrations of melatonin and DIECA in the nutrient solution were set at 10 and 50 μM, respectively. To assess the involvement of melatonin in MeJA-induced FON2 resistance, *p*-chlorophenylalanine (CPA, a melatonin biosynthesis inhibitor) were used to block melatonin biosynthesis (Li et al., 2021). The final concentrations of MeJA and CPA in the nutrient solution were set at 1 and 100 μM, respectively. After a period of 24 h, the roots were inoculated with FON2 spore suspension. Watermelon *caffeic acid O-methyltransferase 1* (*ClCOMT1*) is a key gene involved in melatonin biosynthesis in watermelon (Chang et al., 2021). To further evaluate the role of melatonin in MeJA-induced FON2 resistance, the *clcomt1* mutants were pretreated with 1 μM MeJA for 24 h prior to FON2 inoculation. The *clcomt1* mutants have been previously characterized in our earlier study (Chang et al., 2025). Each treatment group consisted of three replicates with twenty seedlings per replicate.

In order to investigate the impact of different concentrations of melatonin and MeJA on FON2 mycelium growth, the solutions of melatonin or MeJA were filtered, sterilized, and added to the PDA liquid medium. The final concentrations of melatonin used were 0 (control), 1, 10, or 50 μM. The final concentrations of MeJA used were 0 (control), 1, 10, 50, or 100 μM. The medium was thoroughly mixed before being poured into Petri dishes with a volume of 20 mL per dish. FON2 were inoculated onto PDA medium at 28°C for 5 days. The mycelial mass of FON2, with a diameter of 6 mm, obtained from the periphery of cultured colonies were placed on medium containing varying concentrations of melatonin or MeJA, with the mycelium facing downwards. Following an incubation period of 5 days at 28°C, the colony diameter was measured and used to calculate the inhibition rate.

### Disease incidence and index analysis

According to the investigation method described by Martyn and Bruton (1989), the diseased plants are categorized into five grades (from 1 to 5), with a susceptibility type defined as ≥3. The disease index and incidence rate can be calculated using Equations 1 and 2, respectively.


(1) $$O=\frac{\sum a_{i}i}{AI}\times 100$$

(2) $$P=\frac{\sum_{i=3}^{5}a_{i}}{A}\times 100\%$$

*O*: disease index; *a_i_*: number of diseased plants at each grade; *i*: value of disease grade; *A*: total number of inoculated plants; *I*: highest disease grade; *P*: disease incidence, %.

### Dry and fresh weight measurement

The seedlings were carefully extracted from the hydroponic container, and any moisture on the root system was absorbed using a paper towel. The shoots and the roots were delicately severed with a blade, and subsequently weighed for their respective fresh weights. Next, the shoots and roots were placed in an oven set at 105°C for 1 h, after which the temperature was adjusted to 80°C for a duration of 48 h to obtain their dry weights.

### Calculation of mycelial growth inhibition rate

The relative mycelial growth inhibition rate is calculated according to Equation 3.


(3) $$T=\frac{R_{CK}-R}{R_{CK}-r}\times 100\%$$

*T*: the relative mycelial growth inhibition rate, %; *R_CK_*: mycelium growth diameter of control, mm; *R*: mycelium growth diameter of treatments, mm; *r*: original mycelium diameter, mm.

### Quantification of melatonin and MeJA

Melatonin extraction was performed according to the method of Pape and Luning (2006). MeJA extraction was performed as described by Pan et al. (2010). Melatonin and MeJA levels were quantified using their respective ELISA kit (China Agricultural University) following the manufacturer’s instructions.

### Assay of defense enzyme activities and lignin content

The protein content was determined according to the method of Said-Fernández et al., (1990). Polyphenol oxidase (PPO) activity was measured as described by Xin et al. (2016). Phenylalanine ammonia lyase (PAL) activity was measured following the method of Dubery and Smit (1994). PPO and PAL activity was expressed as activity per gram fresh weight. For the determination of lignin content, the leaf samples were dried at 80°C to constant weight, pulverized and passed through a 50-mesh sieve. The lignin content was determined using a lignin assay kit (Solarbio, China) according to the instructions provided by the manufacturer. The lignin content was expressed as the weight percentage of dry weight.

### Quantitative real-time PCR

The watermelon roots were collected for total RNA extraction using the RNA simple Total RNA Kit (TIANGEN, Beijing, China). Following gDNase treatment to eliminate DNA contamination, 1 μg of total RNA was reverse transcribed using a FastKing RT kit (TIANGEN, Beijing, China). qRT-PCR analysis was performed on a StepOnePlus™ Real-Time PCR System (Applied Biosystems, USA) with the SYBR^®^Premix ExTaq™ II (2×) kit (Takara, Tokyo, Japan), following a previously established protocol (Li et al., 2017). Relative expression levels were normalized to watermelon *β-ACTIN*, and calculated using the 2^-ΔΔCT^ method according to Livak and Schmittgen (2001). The gene-specific primers were designed as follows: 5’-CCATGTATGTTGCCATCCAG-3’ and 5’-GGATAGCATGGGGTAGAGCA-3’ for *β-ACTIN* (Cla007792); 5’-CATCCACTAGTCGTCGGTTCCT-3’ and 5’-CCATTCAGGGTCGCTTCTTTGC-3’ for *LOX1* (Cla019897); 5’-AGGTCAAGTTGCAGCAGATCGT-3’ and 5’-AATGGCGGCAGAAGGTTCACAA-3’ for *AOC1* (Cla003512); and 5’-TCGCCACCAAGGGAGTCATTCA-3’ and 5’-GCACAGCAGTGGACCTTGAAAC-3’ for *COMT1* (Cla97C07G144540); 5’-GCGCCTTCTTGAGCAACCAA-3’ and 5’-GGCCATCCTCCAGTAGTCTC-3’ for *pathogenesis-related protein 3* (*PR3*, Cla97C01G020270).

### Statistical analysis

The experiment was conducted following a completely randomized design with three independent biological replicates. Statistical analysis was performed using analysis of variance (ANOVA), and *P* values < 0.05 were considered statistically significant based on Tukey’s test.

## Results

### Melatonin enhances FON2 resistance in watermelon plants

**Figure 1:**
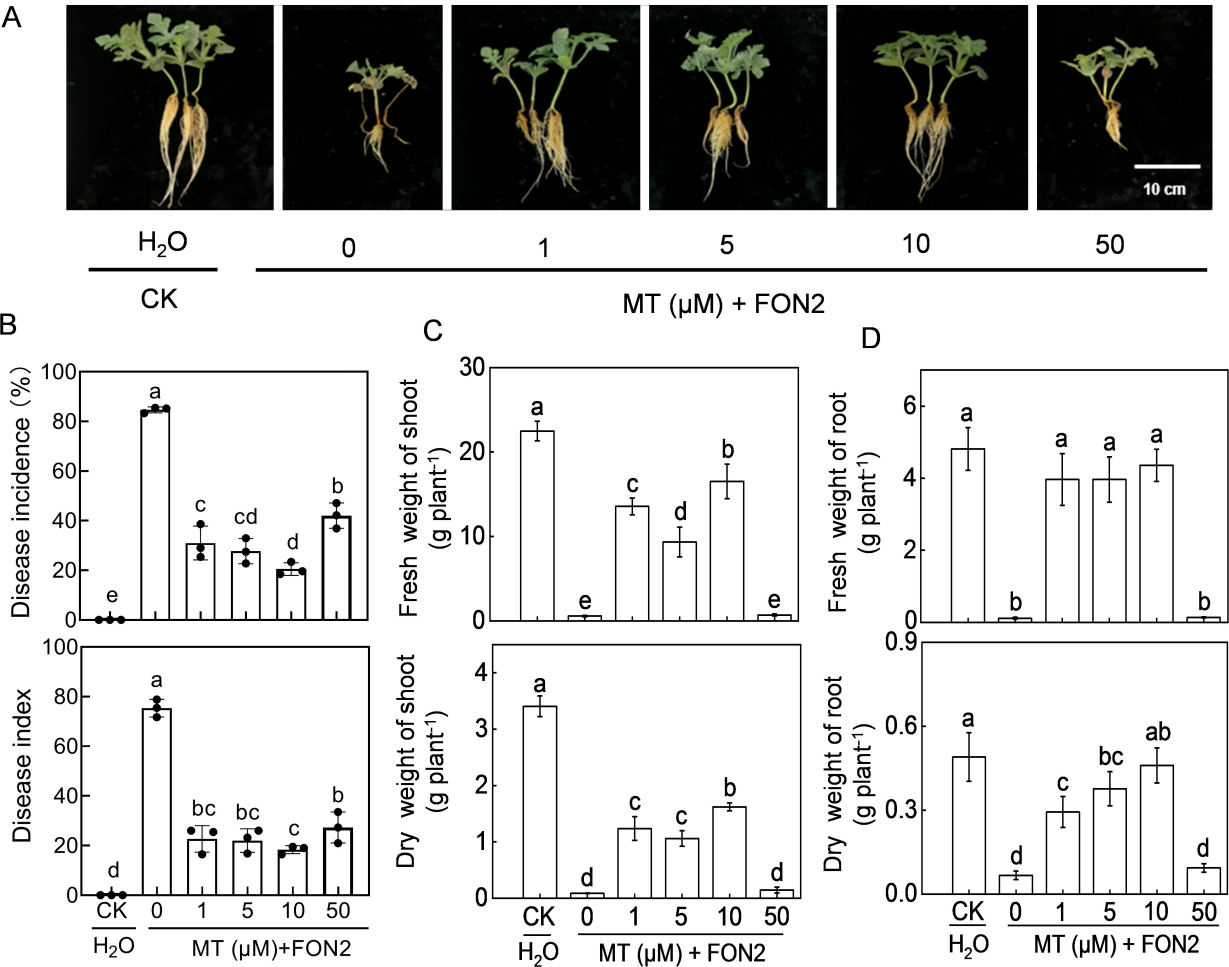
Effects of melatonin on watermelon resistance to *Fusarium oxysporum* f. sp. *Niveum* race 2 (FON2). At the one-leaf stage, seedlings were transplanted into a container filled with Hoagland’s nutrient solution. Three days later, varying concentrations of melatonin (MT) were introduced into the hydroponic nutrient solution, with final concentrations of 0 (CK, control), 1, 5, 10, or 50 μM. After a 24-h period, the seedlings were inoculated with FON2 and maintained for a duration of 11 days. **(A)** Plant phenotype. **(B)** Disease incidence and index (n=3 replicates, 20 plants per replicate). **(C)** Fresh and dry weight of shoots (n=6). **(D)** Fresh and dry weights of roots (n=6). In **(B-D)**, data are expressed as means ± SDs, with significant differences among means indicated by different letters (*p* < 0.05, Tukey’s test).

We first analyzed the effects of melatonin at different concentrations on FON2 resistance of watermelon seedlings. After 11 days of FON2 inoculation, watermelon seedlings exhibited characteristic symptoms of *Fusarium* wilt, including browning and constriction at the stem base, a dark brown root system with extensive rotting, complete desiccation and abscission of nearly all leaves, while only a few plants retained intact foliage (**Figure 1A**). The untreated melatonin plants exhibited an incidence and disease index of 84.7% and 75.3%, respectively (**Figure 1B**). Exogenous melatonin at appropriate dose (1–50 μM) significantly ameliorated the blight symptoms and reduced the incidence and disease index caused by FON2 infection. The most effective concentration of melatonin was determined to be 10 μM. Treatment with 10 μM melatonin resulted in a significant reduction of blight incidence and disease index by 75.8% compared to untreated plants. The protective effect of melatonin against FON2 was attenuated with melatonin concentrations both lower and higher than 10 μM. Compared to control plants, both the shoots and roots of FON2-inoculated plants exhibited a significant reduction in dry weights and fresh weights (**Figures 1C, D**). However, the inhibitory effect of FON2 on plant growth was significantly alleviated by low concentrations (1-10 μM) of melatonin, with an optimal concentration of 10 μM. Conversely, high concentration (50 μM) of melatonin did not exhibit any significant impact on the growth inhibition caused by FON2 infection.

**Figure 2:**
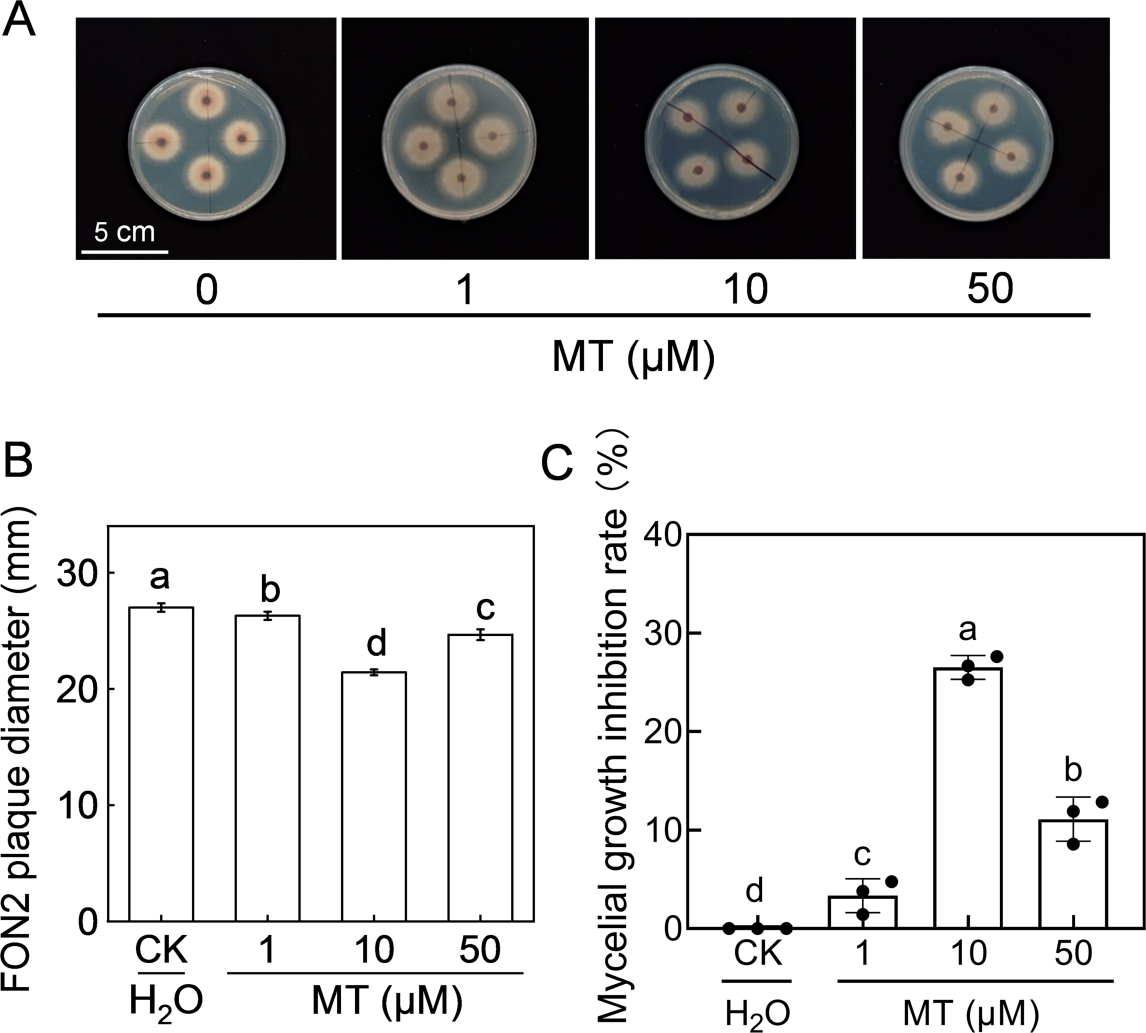
Effects of melatonin on the mycelial growth of *Fusarium oxysporum* f. sp. *Niveum* race 2 (FON2). The melatonin (MT) solutions supplemented into the PDA liquid medium, with final concentrations of 0 (CK, control), 1, 10, or 50 μM. The mycelial mass of FON2, with a diameter of 6 mm, were placed on medium containing varying concentrations of melatonin, with the mycelium facing downwards. Following an incubation period of 5 days, the colony diameter was measured and used to calculate the inhibition rate. **(A)** Mycelial growth on medium. **(B)** FON2 plaque diameter (n=3). **(C)** Mycelial growth inhibition rate (n=3). In **(B, C)**, data are expressed as means ± SDs, with significant differences among means indicated by different letters (*p* < 0.05, Tukey’s test).

The effect of melatonin on FON2 mycelial growth was investigated by inoculating FON2 mycelia onto PDA medium supplemented with varying concentrations (1-50 μM) of melatonin. The colonies grown on the melatonin-supplemented medium exhibited a reduced diameter compared to the control, and the development of aerated mycelia was also less pronounced (**Figures 2A, B**). Notably, 10 μM melatonin exhibited the most effective inhibition against FON2, resulting in a mycelia growth inhibition rate of 26.5% (**Figure 2C**). Conversely, when the concentration of melatonin was increased to 50 μM, the inhibitory effect against FON2 decreased, indicating that the inhibitory effect of melatonin on FON2 mycelial growth is dose-dependent.

### MeJA is involved in melatonin-induced FON2 resistance in watermelon plants

**Figure 3:**
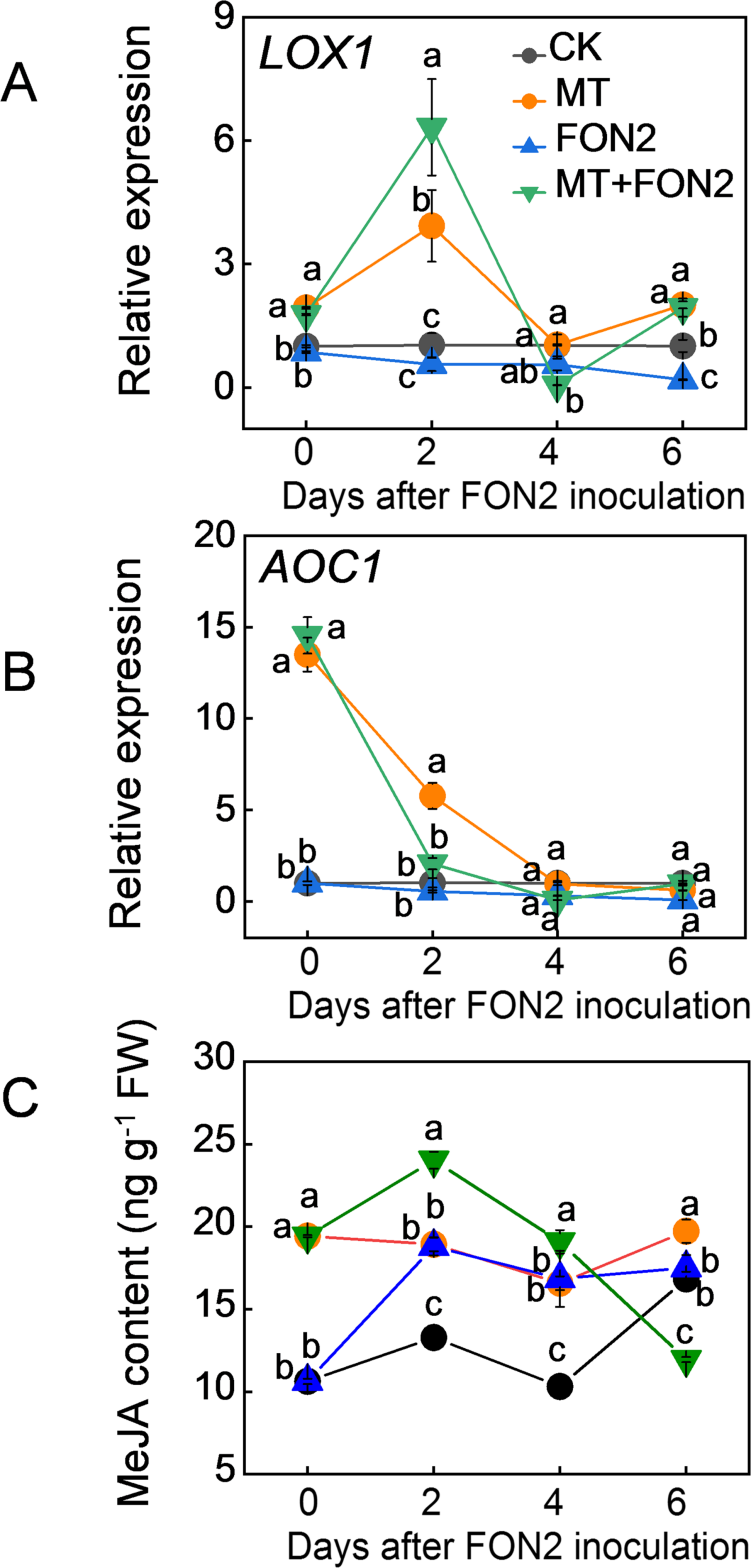
Effects of melatonin on methyl jasmonate (MeJA) biosynthesis in response to *Fusarium oxysporum* f. sp. *Niveum* race 2 (FON2) infection. The seedlings were subjected to the experimental procedures described in **Figure 1**. The hydroponic nutrient solution was supplemented with melatonin (MT) at a final concentration of 10 μM. **(A, B)** The relative expression levels of *lipoxygenases 1* (*LOX1*) and *allene oxide cyclase 1* (*AOC1*), respectively, two pivotal genes involved in MeJA biosynthesis. **(C)** MeJA contents in watermelon roots. Data are expressed as means ± SDs (n=3), with significant differences among means indicated by different letters (*p* < 0.05, Tukey’s test).

To investigate the involvement of MeJA in melatonin-mediated regulation of FON2 resistance in watermelon seedlings, we initially examined the impact of melatonin on MeJA levels as well as the expression of key genes involved in its synthesis. Remarkably, exogenous melatonin significantly stimulated the upregulation of MeJA synthesis genes *LOX1* and *AOC1* both under normal conditions and upon FON2 inoculation (**Figures 3A, B**). The peak expression levels for *LOX1* were observed on the second day post-inoculation, while *AOC1* exhibited high basal expression prior to inoculation followed by a rapid decline thereafter. Inoculation with FON2 resulted in an increase in MeJA content, and the presence of melatonin during early stages of FON2 stress further augmented this elevation (**Figure 3C**). Within four days after FON2 inoculation, plants treated with melatonin exhibited significantly higher levels of MeJA content compared to untreated plants. These results suggest that melatonin induces the synthesis of MeJA during the early and middle stages of FON2 infection.

**Figure 4:**
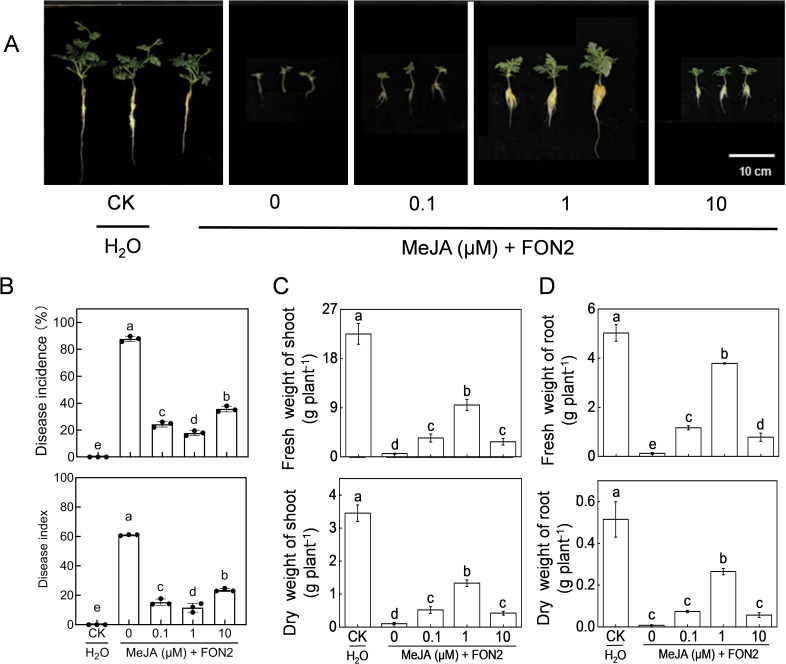
Effects of methyl jasmonate (MeJA) on watermelon resistance to *Fusarium oxysporum* f. sp. *Niveum* race 2 (FON2). At the one-leaf stage, seedlings were transplanted into a container filled with Hoagland’s nutrient solution. Three days later, varying concentrations of MeJA were introduced into the hydroponic nutrient solution for watermelon seedlings, with the final melatonin concentrations set at 0 (CK, control), 0.1, 1, or 10 μM. After a 24-h period, the seedlings were inoculated with FON2 and maintained for a duration of 11 days. **(A)** Plant phenotype. **(B)** Disease incidence and index (n=3 replicates, 20 plants per replicate). **(C)** Fresh and dry weight of shoots (n=6). **(D)** Fresh and dry weight of roots (n=6). In **(B-D)**, data are expressed as means ± SDs, with significant differences among means indicated by different letters (*p* < 0.05, Tukey’s test).

We subsequently examined the impact of varying concentrations of MeJA on watermelon seedling resistance to FON2. Exogenous application of MeJA at appropriate doses (0.1-10 μM) significantly mitigated blight symptoms and reduced the incidence and disease index caused by FON2 infection (**Figures 4A, B**). The most effective concentration of MeJA was determined to be 1 μM. Treatment with 1 μM MeJA resulted in a substantial reduction in both blight incidence (70.0%) and disease index (50.3%), compared to untreated plants. The protective effect of MeJA against FON2 was compromised when using concentrations lower or higher than 1 μM. Notably, at a concentration of 50 or 100 μM, the protective effect of MeJA was completely lost (**Supplementary Figure S1**). Furthermore, MeJA effectively alleviated the inhibitory effect of FON2 on plant growth, with an optimal concentration of 1 μM (**Figures 4C, D**).

**Figure 5:**
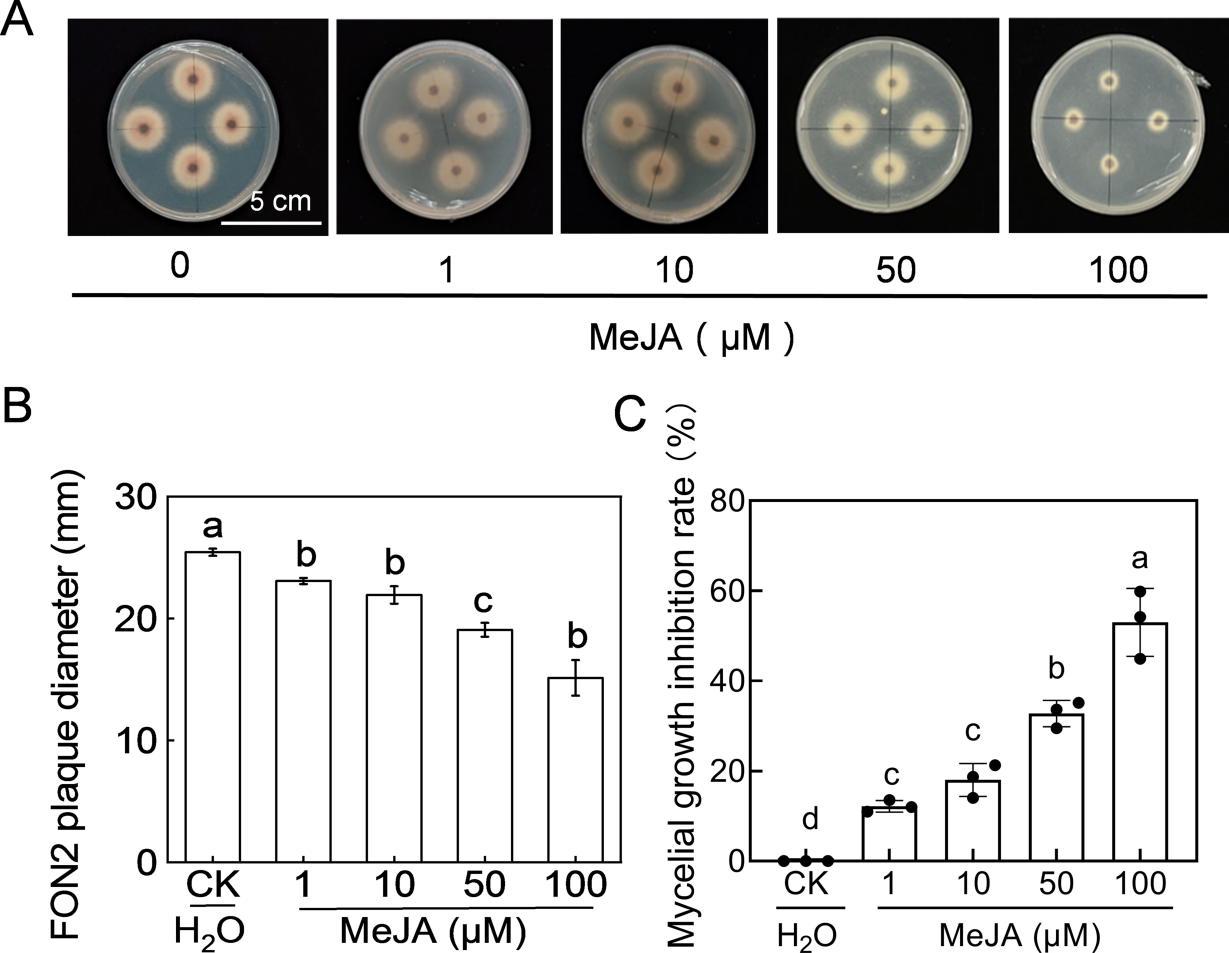
Effects of methyl jasmonate (MeJA) on the mycelial growth of *Fusarium oxysporum* f. sp. *Niveum* race 2 (FON2). The MeJA solutions supplemented into the PDA liquid medium, with final concentrations of 0 (CK, control), 1, 10, 50, and 100 μM. The mycelial mass of FON2, with a diameter of 6 mm, were placed on medium containing varying concentrations of MeJA, with the mycelium facing downwards. Following an incubation period of 5 days, the colony diameter was measured and used to calculate the inhibition rate. **(A)** Mycelial growth on medium. **(B)** FON2 plaque diameter (n=3). **(C)** Mycelial growth inhibition rate (n=3). In **(B, C)**, data are expressed as means ± SDs, with significant differences among means indicated by different letters (*p* < 0.05, Tukey’s test).

The fungus FON2 was inoculated on PDA medium supplemented with varying concentrations (1-100 μM) of MeJA to investigate the impact of MeJA on FON2 mycelial growth. As depicted in **Figures 5A, B**, the diameter of FON2 plaques on MeJA-supplemented PDA medium was significantly smaller than that of the control, and decreased progressively with increasing MeJA concentration. The inhibitory effect of MeJA on FON2 mycelium growth exhibited a dose-dependent relationship, becoming more pronounced as the concentration of MeJA increased (**Figure 5C**). Specifically, inhibition rates for mycelial growth at 1 μM, 10 μM, 50 μM and 100 μM MeJA were determined to be 12.2%, 18.0%, 32.76% and 53.0%, respectively.

**Figure 6:**
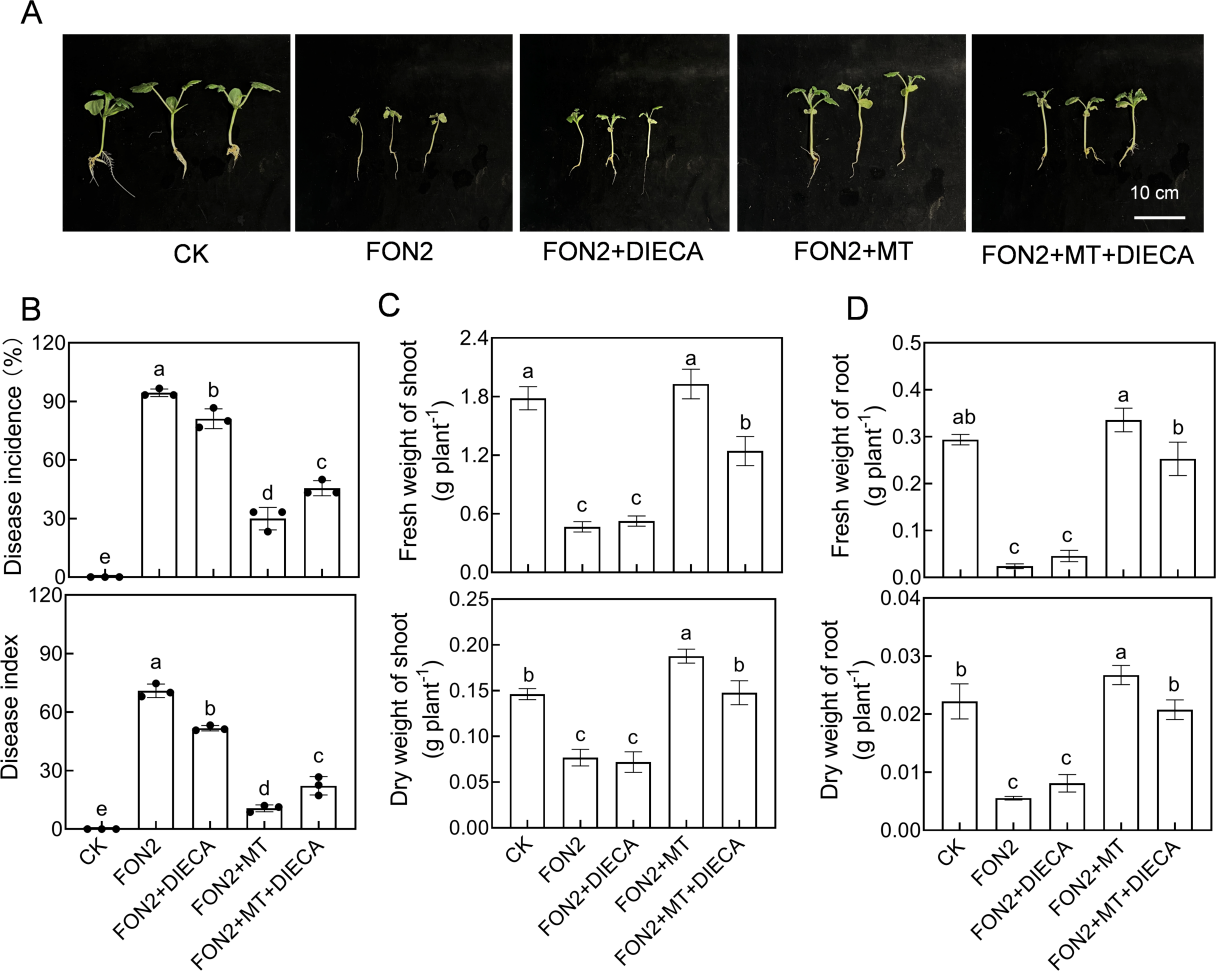
Involvement of methyl jasmonate (MeJA) in melatonin-induced *Fusarium oxysporum* f. sp. *Niveum* race 2 (FON2) resistance in watermelon. At the one-leaf stage, seedlings were transplanted into a container filled with Hoagland’s nutrient solution. Three days later, melatonin or a combination of melatonin (MT) and diethyldithiocarbamic acid (DIECA, a JA biosynthesis inhibitor) was introduced into the hydroponic nutrient solution at final concentrations of 10 μM and 50 μM for melatonin and DIECA, respectively. After a 24-h period, the seedlings were inoculated with FON2 and maintained for a duration of 11 days. **(A)** Plant phenotype. **(B)** Disease incidence and index (n=3 replicates, 20 plants per replicate). **(C)** Fresh and dry weights of shoots (n=6). **(D)** Fresh and dry weights of roots (n=6). In **(B-D)** data are expressed as means ± SDs, with significant differences among means indicated by different letters (*p* < 0.05, Tukey’s test).

To further investigate the involvement of MeJA in regulating melatonin-mediated resistance to FON2 in watermelon seedlings, we examined the impact of a MeJA synthesis inhibitor (DIECA) on melatonin’s regulation of FON2 resistance in watermelon. Treatment with DIECA (50 μM) suppressed the induction of melatonin-induced resistance against FON2 (**Figures 6A, B**). Moreover, compared to the plants treated with melatonin+FON2, those treated with melatonin+DIECA+FON2 exhibited a significant reduction of 21.4% and 35.8% in shoot dry weight and fresh weight, respectively, while root dry weight and fresh weight decreased by 22.3% and 24.7%, respectively (**Figures 6C, D**). These findings suggest that MeJA plays a crucial role in mediating melatonin-induced resistance against FON2 infection in watermelon seedlings.

### MeJA feedback enhances melatonin accumulation in response to FON2 infection

**Figure 7:**
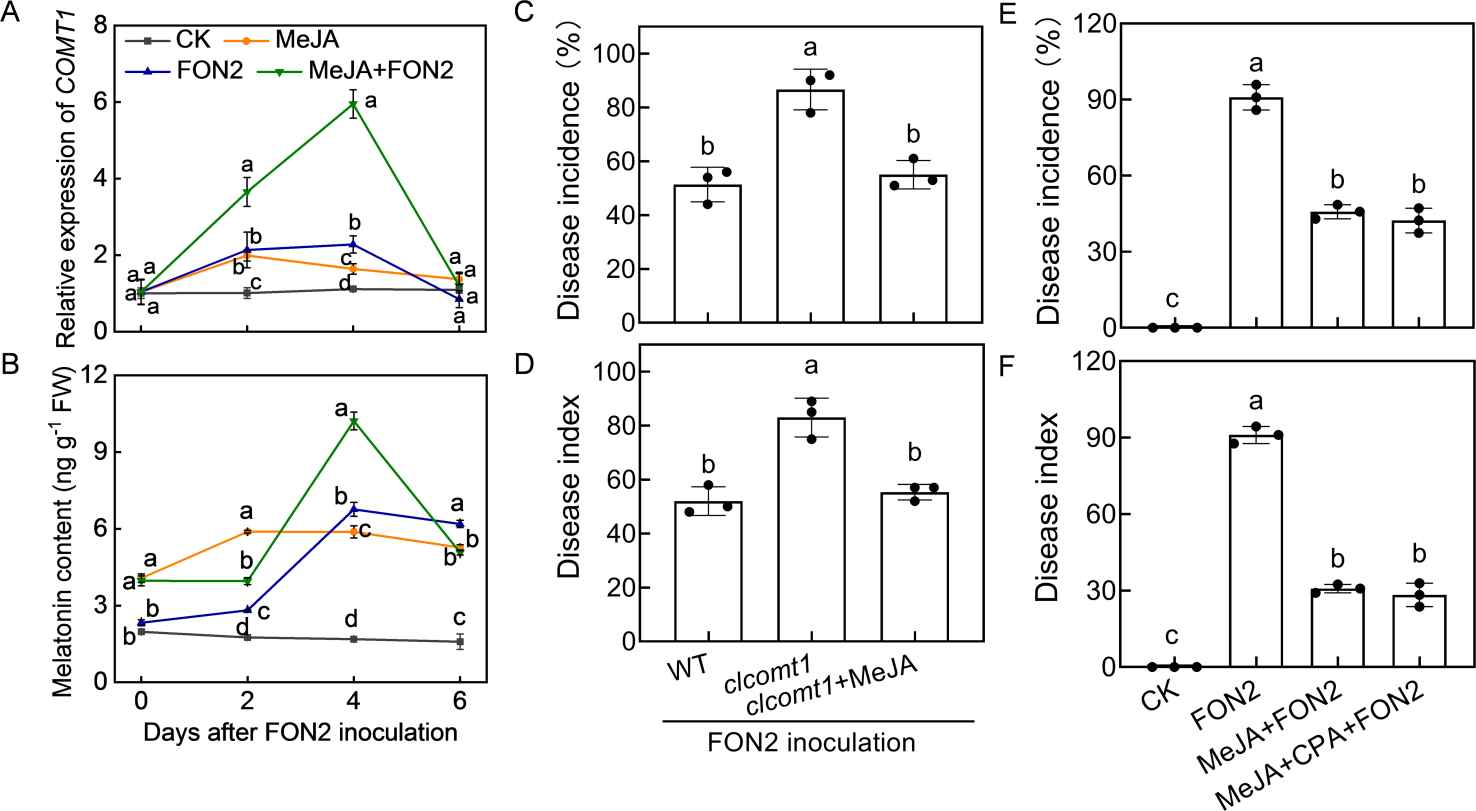
Methyl jasmonate (MeJA) feedback promotes melatonin accumulation in response to *Fusarium oxysporum* f. sp. *Niveum* race 2 (FON2) infection. **(A)** The relative expression levels of *caffeic acid O-methyltransferase 1* (*COMT1*), a pivotal gene involved in melatonin biosynthesis. **(B)** Melatonin contents in watermelon roots. The seedlings were subjected to the experimental procedures described in **Figure 4**. The hydroponic nutrient solution was supplemented with MeJA at a final concentration of 1 μM. **(C, D)** Disease incidence and Disease index. The *clcomt1* were pretreated with 1 μM MeJA for 24 h prior to FON2 inoculation. **(E, F)** Disease incidence and Disease index. At the one-leaf stage, seedlings were transplanted into a container filled with Hoagland’s nutrient solution. Three days later, MeJA or a combination of MeJA and *p*-chlorophenylalanine (CPA, a melatonin biosynthesis inhibitor) was introduced into the hydroponic nutrient solution at final concentrations of 1 μM and 100 μM for MeJA and CPA, respectively. After a 24-h period, the seedlings were inoculated with FON2 and maintained for a duration of 11 days. Data are expressed as means ± SDs (n=3), with significant differences among means indicated by different letters (*p* < 0.05, Tukey’s test). *clcomt1*, watermelon *caffeic acid O-methyltransferase 1*.

To assess whether MeJA regulates melatonin biosynthesis in a feedback manner, we examined the impact of MeJA on melatonin biosynthesis. The application of MeJA significantly enhanced the relative expression of *caffeic acid O-methyltransferase 1* (*COMT1*), a pivotal gene involved in melatonin biosynthesis, and promoted melatonin accumulation in watermelon roots both under normal conditions and upon FON2 infection (**Figures 7A, B**). Following four days of FON2 infection, the mRNA abundance of *COMT1* and melatonin levels in melatonin-pretreated plants exhibited peaks that were 161% and 51% higher, respectively, compared to those observed in plants without melatonin treatments. Moreover, the deletion of *ClCOMT1* significantly reduced watermelon resistance against FON2; however, this reduction in resistance was fully restored through exogenous application of MeJA (**Figures 7C, D**). Additionally, inhibition of melatonin biosynthesis by CPA pretreatment did not significantly affect the MeJA-induced FON2 resistance (**Figures 7E, F**). Furthermore, the protective effect of melatonin+MeJA combination was comparable to that of MeJA alone (**Supplementary Figure S2**). Taken together, these results suggest that melatonin-induced MeJA promotes melatonin accumulation in a feedback loop, forming a reciprocal positive-regulatory loop that enhances watermelon resistance against FON2.

**Figure 8:**
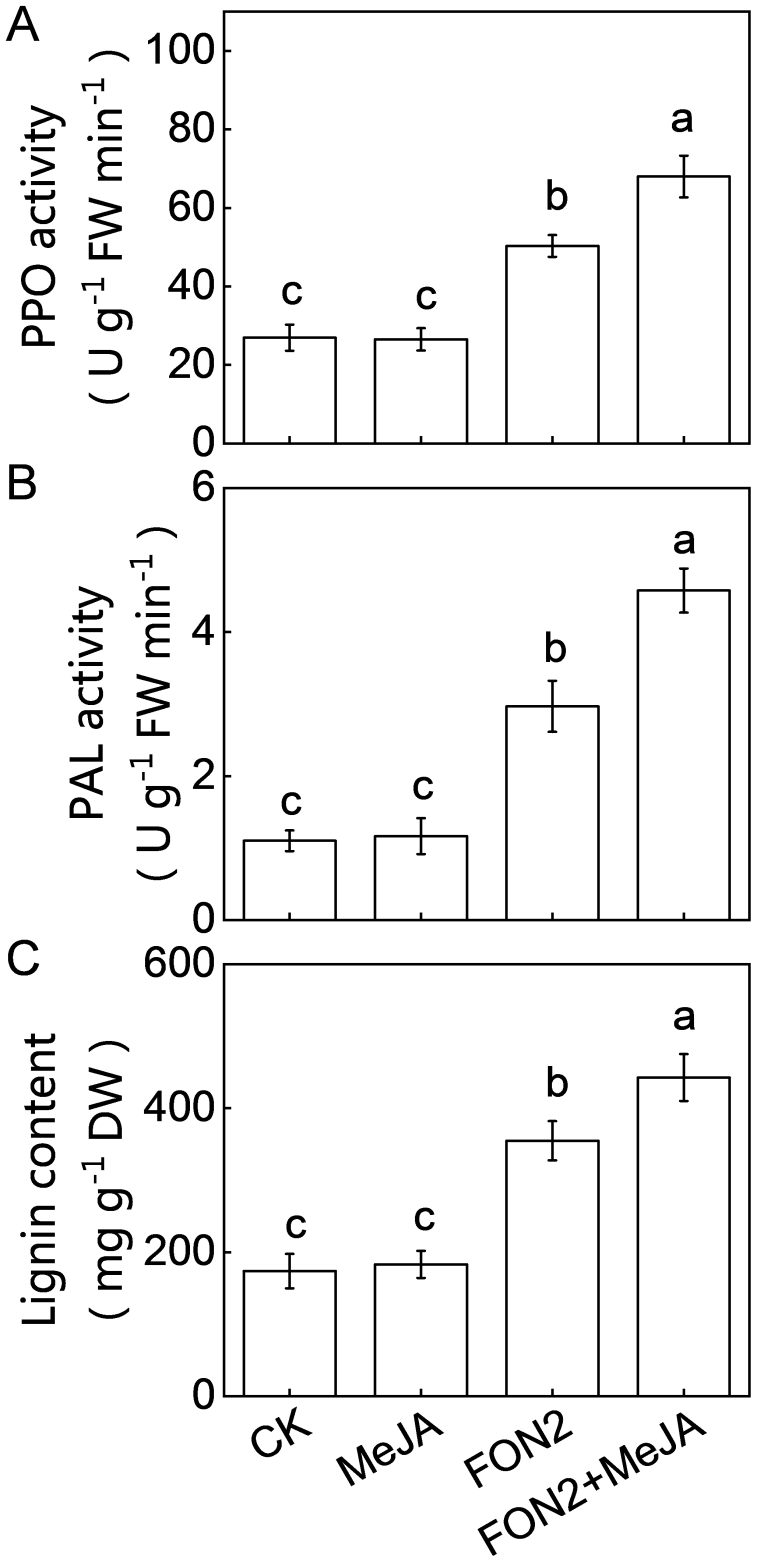
Effects of methyl jasmonate (MeJA) on the activities of defense-related enzymes and lignin content in response to *Fusarium oxysporum* f. sp. *Niveum* race 2 (FON2) infection. The seedlings were subjected to the experimental procedures described in **Figure 7A**. **(A)** Polyphenol oxidase (PPO) activity. **(B)** Phenylalanine ammonia lyase (PAL) activity. **(C)** Lignin content. Data are expressed as means ± SDs (n=3), with significant differences among means indicated by different letters (*p* < 0.05, Tukey’s test).

Lignin and defense-related enzymes, such as PPO and PAL play critical roles in plants defense against fungal pathogens. Under normal conditions, MeJA pretreatment did not significantly alter the activities of PPO and PAL or the lignin content (**Figures 8A–C**). However, upon FON2 infection, the activities of PPO and PAL, as well as the lignin content, were significantly elevated, with these increases being further enhanced by exogenous MeJA application. Specifically, on the second day post-FON2 inoculation, the activities of PPO and PAL and the lignin content in MeJA-pretreated plants were 35.2%, 54.2%, and 24.8% higher, respectively, compared to those in unpretreated plants. These results suggest that PPO, PAL, and lignin may be key components in the melatonin- and MeJA-induced resistance against FON2. Furthermore, pathogenesis-related (PR) proteins are crucial for plant defense against pathogens. Our results show that melatonin, MeJA, and their combination significantly upregulated the expression of *PR3* under FON2 stress (**Supplementary Figure S3**).

## Discussion

Melatonin, a potent antioxidant and stress tolerance inducer, is increasingly studied for its potential in enhancing plant resistance against various fungal diseases. These include potato late blight caused by *Phytophthora infestans* (Zhang et al., 2017), powdery mildew in watermelon caused by *Podosphaera xanthii* and crown rot in cucurbits caused by the soilborne fungus *Phytophthora capsici* (Mandal et al., 2018), gray mold in cherry tomato caused by *Botrytis cinerea* (Li et al., 2019a), anthracnose in banana caused by *Colletotrichum musae* (Li et al., 2019b), tomato fruit decay caused by *Botrytis cinerea* (Liu et al., 2019), downy mildew in cucumber caused by *Pseudoperonospora cubensis* (Sun et al., 2019) as well as *Fusarium* wilt caused by *Fusarium oxysporum* f. sp. *cucumerinum* in cucumber (Ahammed et al., 2020). Consistent with previous studies, our data demonstrates that the exogenous application of melatonin enhances watermelon resistance against FON2 infection in a dose-dependent manner (**Figure 1**). However, both higher and lower concentrations of melatonin than the optimal concentration (around 10 μM) attenuate its protective effect.

Melatonin has been reported to effectively inhibit cell growth in certain human pathogens by impairing their mitochondrial functions, inhibiting biofilm formation, and reducing intracellular substrates (Tekbas et al., 2008; Elmahallawy et al., 2014; Yang et al., 2014). Studies have explored the indirect effects of melatonin on plant pathogens through triggering plant immunity. However, the direct interaction between melatonin and plant pathogens remains poorly understood (Arnao and Hernández-Ruiz, 2015). Qu et al. (2022) found no significant inhibition of mycelial growth in *Alternaria alternata*, *Botrytis cinerea*, and *Colletotrichum gloeosporioides* when treated with melatonin. Li et al. (2019a) also found that melatonin did not exhibit *in vitro* antifungal activity against *Botrytis cinerea.* Nevertheless, exogenous melatonin significantly attenuated potato late blight by inhibiting mycelial growth of *Phytophthora infestans* (Zhang et al., 2017). In the current study, melatonin inhibited FON2 mycelial growth on PDA medium in a dose-dependent manner, and such inhibitory effect was attenuated with melatonin concentrations both lower and higher than 10 μM (**Figure 2**). The diminished inhibitory effect at higher melatonin concentrations may be attributable to the adaptive mechanisms of FON2 cells that counteract the suppressive effects of melatonin. These findings suggest that melatonin not only regulates plant defense response but also exhibits potential in mitigating FON2-induced damage by inhibiting FON2 growth in plants to a certain extent.

In addition to disrupting the vascular system and thereby impeding water and nutrient uptake, Fo inoculation also significantly diminished photosynthetic activity, resulting in the inhibition of seedling growth (Ahammed et al., 2020). In the current study, the reduction in dry weight and fresh weight of seedlings caused by FON2 infection was partially attributed to the inhibition of photosynthetic activity (**Figures 1C, D**, **4C, D**, **6C, D**). Notably, melatonin can improve photosynthetic carbon assimilation possibly by improving stomatal conductance and Calvin cycle enzymes, which may lead to an increase in plant weight (Ding et al., 2017; Ahammed et al., 2020; Wang et al., 2022). Therefore, it can be inferred that melatonin mitigates the inhibitory effect of FON2 on seedling growth by suppressing FON2 infection, while simultaneously enhancing seedling resistance to FON2 through the improvement of photosynthetic activity and promotion of seedling growth.

Increasing evidence has demonstrated the pivotal regulatory role of the JA signaling pathways in plant defense against fungal diseases, such as fusarium wilt (Wang et al., 2021). However, it is noteworthy that JA signaling can exhibit contrasting effects on host resistance and Fo pathogenicity in different host-Fo interactions (Fernandes and Ghag, 2022). For instance, IbBBX24 promotes sweet potato resistance against Fo through activation of the JA pathway (Zhang et al., 2020), while deletion of the JA synthesis gene *def1* enhances tomato’s susceptibility to Fo infection (Kavroulakis et al., 2007). In *Arabidopsis*, it has been observed that mutations in *coi1* and other JA receptor genes enhance plant resistance against certain Fo pathogens (Thatcher et al., 2009). Notably, mutations in Arabidopsis’ JA synthesis genes, namely *allene oxide synthase* (*aos*) and *12-oxophytodienoate reductase3* (*opr3*), do not significantly impact Fo resistance levels (Thatcher et al., 2009). The influence exerted by JA on Fo resistance may be contingent upon specific pathogenesis mechanisms employed by different strains or species of Fo. Here, our data showed that treatment with MeJA induced a dose-dependent resistance of watermelon against FON2, with an optimal concentration observed at approximately 1 μM (**Figure 4**). Additionally, similar to melatonin, MeJA also exhibited a dose-dependent inhibition of FON2 mycelial growth on PDA medium (**Figure 5**), indicating that MeJA suppresses FON2 infection by impeding its growth in plants and inducing plant defense responses.

JA interacts with other plant hormones to modulate plant stress resistance (Zhao et al., 2021a, b). Emerging evidence highlights the cross talk between melatonin and JA in plant response to various abiotic and biotic stresses (Huang and Jin, 2024). For instance, MeJA regulates melatonin-induced low temperature resistance in grafted watermelon through the modulation of H_2_O_2_ signaling (Li et al., 2021). Melatonin activates the MeJA signaling pathway to enhance the resistance of tomato fruit against *Boturea cineraria* (Liu et al., 2019). Furthermore, melatonin improves the resistance of JA-insensitive mutants of *Arabidopsis* against the virulent bacterium *Pseudomonas syringae* (Liu et al., 2021). Additionally, the JA signaling pathway contributes to melatonin-enhanced the postharvest disease resistance in blueberries fruit (Qu et al., 2022). Recently, it has been reported that MeJA mediates melatonin-induced defense response and subsequent aphid resistance of watermelon via H_2_S signaling (Su et al., 2023). However, the crosstalk between melatonin and MeJA in Fo responses remains largely unknown. In this study, treatment with melatonin resulted in an increased accumulation of MeJA upon FON2 infection (**Figure 3**). Inhibition of MeJA biosynthesis by DIECA pretreatment attenuated the melatonin-induced FON2 resistance (**Figure 6**). Furthermore, the application of MeJA led to an enhanced accumulation of melatonin in watermelon roots upon FON2 infection (**Figures 7A, B**). Notably, the diminished resistance to FON2 caused by *ClCOMT1* deletion was completely restored through exogenous MeJA treatment (**Figures 7C, D**). Moreover, inhibition of melatonin biosynthesis by CPA pretreatment did not significantly affect the MeJA-induced FON2 resistance (**Figures 7E, F**). These findings indicate that melatonin promotes MeJA accumulation, thereby activating JA signaling pathways, such as the JAZ-MYC2 module, and consequently enhancing resistance to FON2 (Zhang et al., 2020). Simultaneously, MeJA provides feedback to enhance melatonin accumulation through upregulating the expression of *ClCOMT1*, thus establishing a reciprocal positive regulatory loop in response to FON2 infection. Moreover, MeJA induced the activities of PPO and PAL, as well as increased lignin content upon FON2 infection, suggesting that PPO, PAL, and lignin play crucial roles in the melatonin- and MeJA- mediated resistance against FON2.

## Conclusions

To date, the signaling mechanisms underlying melatonin-induced Fo resistance of plants are still unclear. This study elucidates a mechanism underlying melatonin-induced FON2 resistance in watermelon. Melatonin inhibits FON2 mycelial growth and suppresses FON2 infection, while also inducing MeJA biosynthesis. MeJA enhances the activities of PPO and PAL, as well as increasing lignin content, thereby impeding FON2 mycelium growth and mitigating the detrimental impact caused by FON2 infection. Moreover, MeJA provides feedback to enhance melatonin accumulation, forming a reciprocal positive regulatory loop in response to FON2 infection. The growing concern over minimizing pesticide usage and transitioning to sustainable and natural control strategies underscores the significant potential of such a mechanism in combating Fo.

## Data Availability

The original contributions presented in the study are included in the article/**Supplementary Material**. Further inquiries can be directed to the corresponding author.
