# Supplementary material for: Positive interaction between melatonin and methyl jasmonate enhances Fusarium wilt resistance in Citrullus lanatus
Source: Front Plant Sci. 2025 May 1;16:1508852. doi: 10.3389/fpls.2025.1508852 (PMC12078211; doi:10.3389/fpls.2025.1508852)
Supplement: Supplementary file 1 [file DataSheet1.docx]

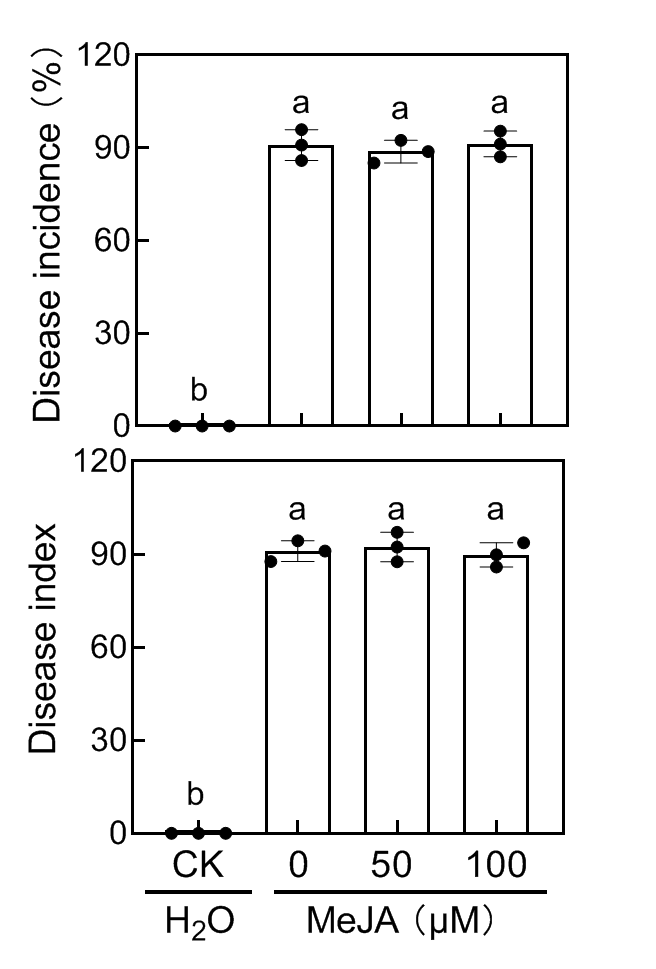


**Supplemental Figure S1.** Effects of methyl jasmonate (MeJA) at 50 and 100 μM on watermelon resistance to *Fusarium oxysporum* f. sp. *Niveum* race 2 (FON2).


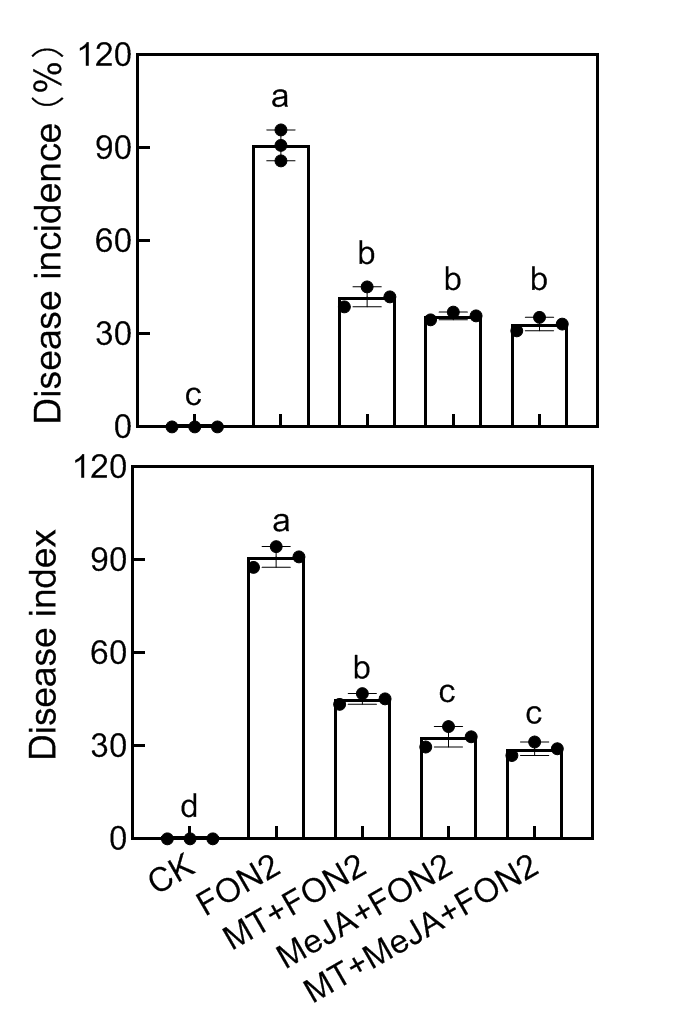


**Supplemental Figure S2.** Effects of melatonin and methyl jasmonate (MeJA) combination on watermelon resistance to *Fusarium oxysporum* f. sp. *Niveum* race 2 (FON2).

**Supplemental Figure S3.** Effects of melatonin, methyl jasmonate (MeJA), and their combination on the expression of *pathogenesis-related protein 3* (*PR3*) under *Fusarium oxysporum* f. sp. *Niveum* race 2 (FON2) stress.
